# Supplementary material for: A Realistic Mixture of Persistent Organic Pollutants Affects Zebrafish Development, Behavior, and Specifically Eye Formation by Inhibiting the Condensin I Complex
Source: Toxics. 2023 Apr 9;11(4):357. doi: 10.3390/toxics11040357 (PMC10146850; doi:10.3390/toxics11040357)
Supplement: Supplementary file 1 [file toxics-11-00357-s001.zip › Figure S1.pptx]

## Slide 1
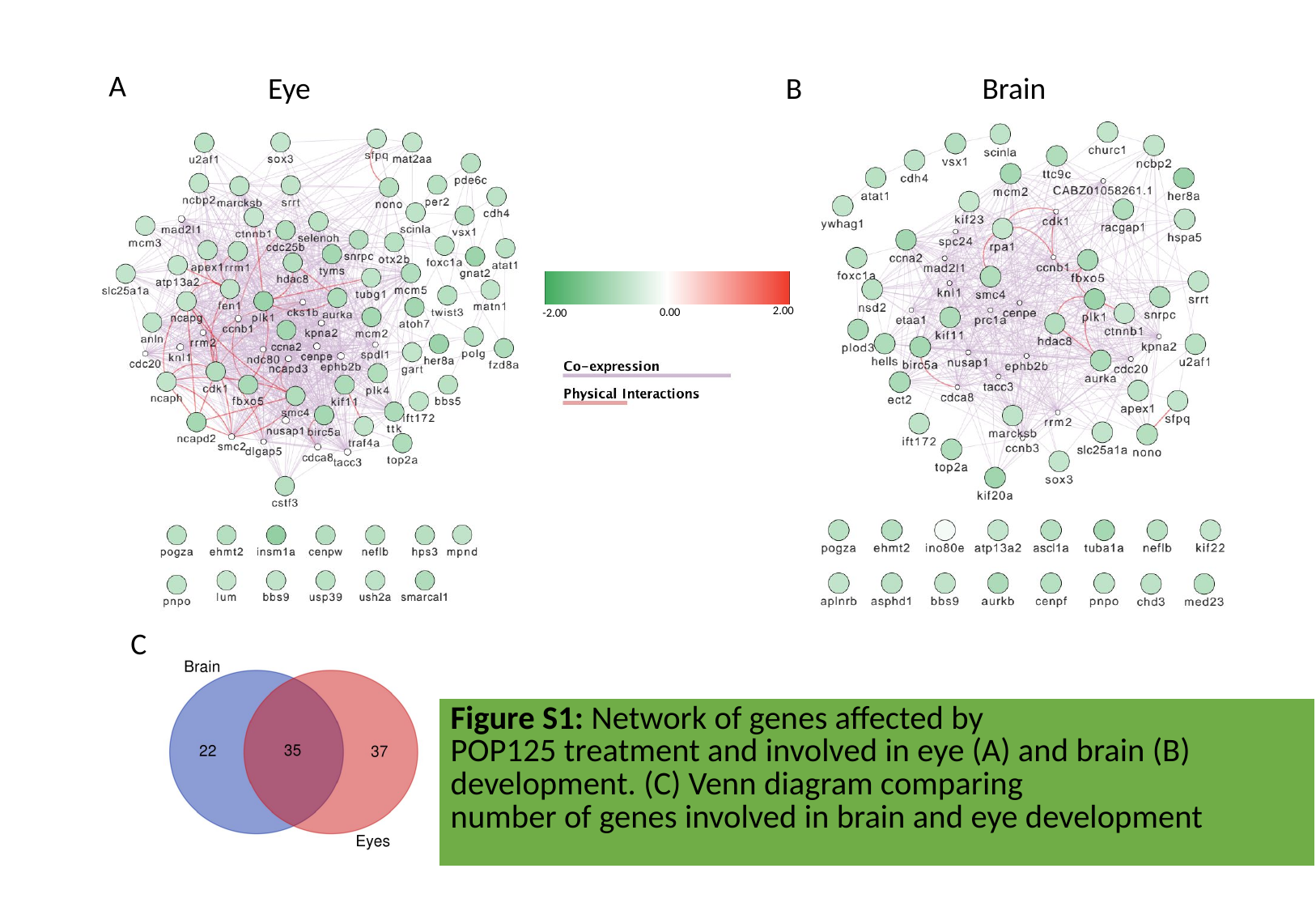

A
Eye
B
Brain
2.00
0.00
-2.00
C
| Figure S1: Network of genes affected by POP125 treatment and involved in eye (A) and brain (B) development. (C) Venn diagram comparing number of genes involved in brain and eye development |
| --- |
