# Supplementary material for: A Realistic Mixture of Persistent Organic Pollutants Affects Zebrafish Development, Behavior, and Specifically Eye Formation by Inhibiting the Condensin I Complex
Source: Toxics. 2023 Apr 9;11(4):357. doi: 10.3390/toxics11040357 (PMC10146850; doi:10.3390/toxics11040357)
Supplement: Supplementary file 1 [file toxics-11-00357-s001.zip › Supplementary table S1.pdf]

Chemical	Measured concentration of stock solution (μM)	Measured concentration of stock solution (mg/ml)
**PFASs**		
PFOA	4,209.4	1.743
PFOS	41,522.1	22.348
PFDA	375.4	0.193
PFNA	1,092.5	0.507
PFHxS	7,809.2	3.422
PFUnDA	336.8	0.190
**Br (Brominated flame retardants)**		
BDE-47	17.8	0.009
BDE-99	7.5	0.004
BDE-100	3.8	0.002
BDE-153	2.1	0.001
BDE-154	3.0	0.002
BDE-209	9.4	0.009
HBCD	54.5	0.035
**Cl mix**		
**PCBs**		
PCB 28	31.1	0.008
PCB 52	20.5	0.006
PCB 101	24.5	0.008
PCB 118	137.9	0.045
PCB 138	429.5	0.155
PCB 153	698.3	0.252
PCB 180	339.0	0.134
**Other organochlorines**		
p,p′-DDE	1,065.9	0.339
HCB	228.2	0.065
α-chlordane	23.7	0.010
Oxy-chlordane	33.0	0.014
Trans-nonachlor	99.1	0.044
α-HCH	16.8	0.005
β-HCH	75.6	0.022
γ-HCH	16.8	0.005
Dieldrin	56.2	0.021

Table S1. List of compounds and concentrations in the POP mix and in the PFAA, Br, and CL sub-mixtures.
